# P-359. Evaluation of Relative Bioavailability and Food Effect of a Fixed-Dose Combination Tablet of Islatravir and Lenacapavir

**DOI:** 10.1093/ofid/ofaf695.577

**Published:** 2026-01-11

**Authors:** Jing Niu, Haeyoung Zhang, John Ling, Sharline Madera, Nerissa Kwok, Steve West, Diane Longo, Gillian Gillespie, Cyril Llamoso, Dhananjay Marathe

**Affiliations:** Gilead Sciences, Inc., Foster City, CA, USA, Foster City, CA; Gilead Sciences Inc, Foster City, California; Gilead Sciences, Inc., Foster City, CA, USA, Foster City, CA; Gilead Sciences, Inc., Foster City, California; Gilead Sciences, Inc., Foster City, CA, USA, Foster City, CA; Gilead Sciences, Inc., Foster City, CA, USA, Foster City, CA; Merck & Co., Inc., Rahway, NJ, USA, Rahway, New Jersey; Merck & Co., Inc., Rahway, NJ, USA, Rahway, New Jersey; Merck & Co., Inc., Rahway, NJ, USA, Rahway, New Jersey; Gilead Sciences, Inc., Foster City, California

## Abstract

**Background:**

Combination treatment with islatravir (ISL), a nucleoside reverse transcriptase translocation inhibitor, and lenacapavir (LEN), an HIV-1 capsid inhibitor, has the potential to provide a complete, once-weekly (QW) oral regimen for HIV-1 treatment. In an ongoing Phase 2 study, 94% of participants receiving QW ISL and LEN co-administration maintained virologic suppression at Week 48. Phase 3 studies are evaluating a fixed-dose combination (FDC) tablet of ISL/LEN. To inform development of the FDC for the Phase 3 studies, a Phase 1 study was conducted to examine the pharmacokinetics (PK) of ISL/LEN FDC vs ISL and LEN single-agent co-administration, and the effect of food on ISL/LEN FDC PK.

**Figure 1.**
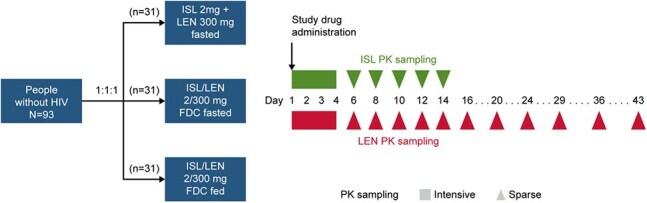
Study Design Safety was assessed at each visit and AEs were recorded through the Day 61 follow-up phone call. PK sampling occurred through Day 14 for ISL and Day 43 for LEN and samples were analyzed by high-performance liquid chromatography–tandem mass spectrometry. AE, adverse event; FDC, fixed-dose combination; ISL, islatravir; LEN, lenacapavir; PK, pharmacokinetics.

**Figure 2.**
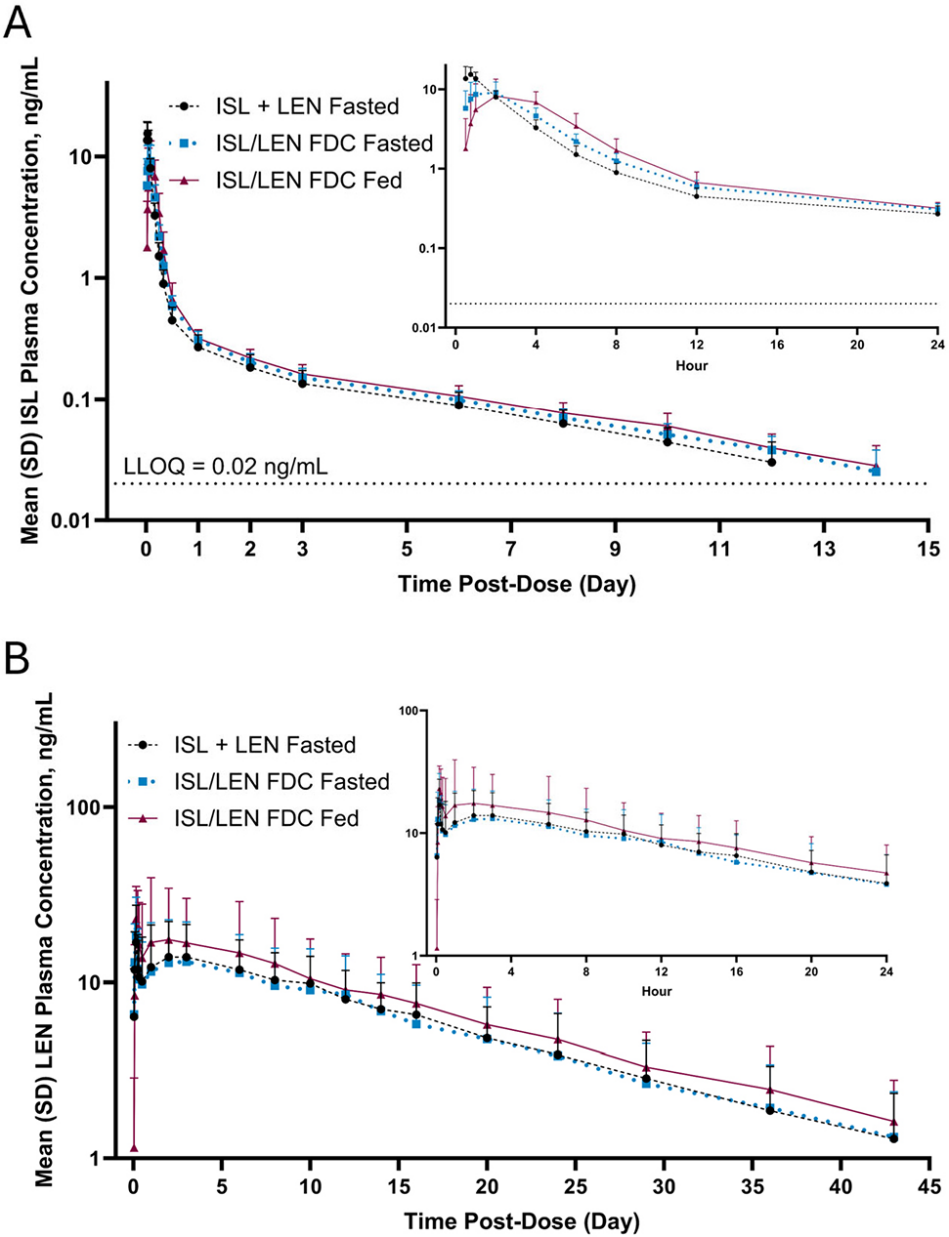
Arithmetic Mean (SD) Plasma Concentration-Time Profiles of (A) ISL and (B) LEN After a Single Dose of ISL and LEN Single-Agent Co-Administration Under Fasted Conditions, and ISL/LEN FDC Under Fasted or Fed Conditions* *First 24 hours shown in insets. The LLOQ for ISL and LEN was 0.02 ng/mL and 0.1 ng/mL, respectively. FDC, fixed-dose combination; ISL, islatravir; LEN, lenacapavir; LLOQ, lower limit of quantitation.

**Methods:**

In this Phase 1, open-label, parallel-design study, participants without HIV-1 received single oral doses of ISL 2 mg and LEN 300 mg single agents simultaneously under fasted conditions (n=31) or ISL/LEN FDC 2/300 mg under fasted (n=31) or fed conditions (n=31) (Figure 1). Plasma PK samples were analyzed using validated methods. PK parameters were determined by standard non-compartmental analysis; geometric least-squares mean ratios were calculated for test vs reference treatment. Safety was monitored by physical examinations, clinical laboratory tests, and adverse event reporting.

**Table 1.**
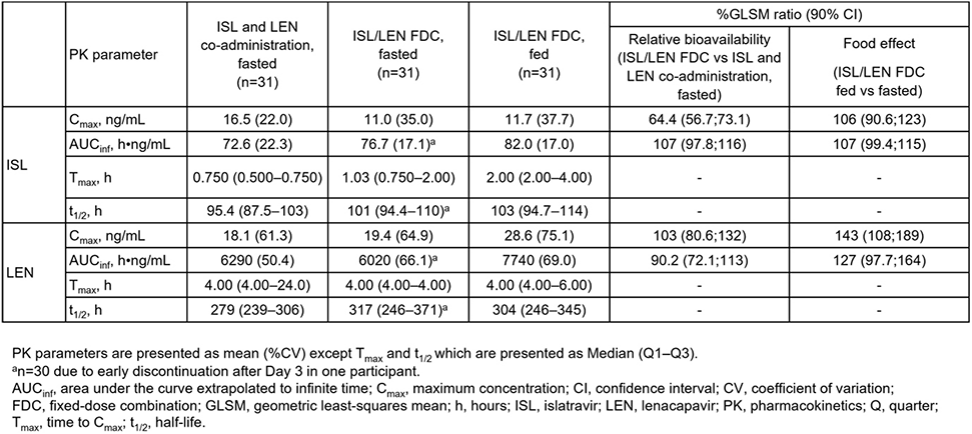
PK Parameter Estimates and Comparisons

**Results:**

ISL and LEN PK parameters were comparable (e.g., extrapolated areas under the concentration-time curves [AUC_inf_] % geometric least squares mean ratios [90% CI]: 107 [97.8; 116] and 90.2 [72.1; 113], respectively) for ISL/LEN FDC vs single-agent co-administration under fasted conditions (Figure 2; Table 1), with the exception of a lower ISL maximum concentration for the FDC. However, this finding is not considered clinically meaningful, as AUC_inf_ indicates a similar extent of ISL absorption with a slower absorption rate. Similar exposures for ISL and elevated exposures for LEN were observed under fed vs fasted conditions for the ISL/LEN FDC (Table 1) that were considered not clinically meaningful. There were no new safety concerns.

**Conclusion:**

The PK results from this relative bioavailability and food-effect characterization of ISL/LEN 2/300 mg FDC support continued clinical development of an ISL/LEN QW oral FDC dosing regimen without regard to food for the treatment of HIV-1 infection.

**Disclosures:**

Jing Niu, MD, MS, Gilead Sciences, Inc.: Employee|Gilead Sciences, Inc.: Stocks/Bonds (Public Company) Haeyoung Zhang, PharmD, PhD, Gilead Sciences, Inc.: Employee|Gilead Sciences, Inc.: Stocks/Bonds (Private Company) John Ling, n/a, Gilead Sciences, Inc.: Employee|Gilead Sciences, Inc.: Stocks/Bonds (Private Company) Sharline Madera, MD, PhD, Gilead Sciences, Inc.: Employee|Gilead Sciences, Inc.: Stocks/Bonds (Public Company) Nerissa Kwok, PharmD, Gilead Sciences, Inc.: Employee Steve West, MSPH, Gilead Sciences, Inc.: Employee|Gilead Sciences, Inc.: Stocks/Bonds (Public Company) Diane Longo, PhD, Merck & Co., Inc.: Employee|Merck & Co., Inc.: Stocks/Bonds (Public Company) Gillian Gillespie, MB, Bchir, Dohme: employee|Dohme: Stocks/Bonds (Public Company)|Merck & Co., Inc.: Employee|Merck & Co., Inc.: Stocks/Bonds (Public Company) Cyril Llamoso, MD, Merck & Co., Inc.: Employee Dhananjay Marathe, PhD, Gilead Sciences, Inc.: Employee|Gilead Sciences, Inc.: Stocks/Bonds (Private Company)

